# Computer-Vision- and Deep-Learning-Based Determination of Flow Regimes, Void Fraction, and Resistance Sensor Data in Microchannel Flow Boiling

**DOI:** 10.3390/s24113363

**Published:** 2024-05-24

**Authors:** Mark Schepperle, Shayan Junaid, Peter Woias

**Affiliations:** Laboratory for the Design of Microsystems, Department of Microsystems Engineering—IMTEK, University of Freiburg, 79110 Freiburg, Germany; schepperle@imtek.de (M.S.); shayanjunaidtalat@gmail.com (S.J.)

**Keywords:** computer vision, convolutional neural network, deep learning, image processing, microchannel flow boiling

## Abstract

The aim of this article is to introduce a novel approach to identifying flow regimes and void fractions in microchannel flow boiling, which is based on binary image segmentation using digital image processing and deep learning. The proposed image processing pipeline uses adaptive thresholding, blurring, gamma correction, contour detection, and histogram comparison to separate vapor from liquid areas, while the deep learning method uses a customized version of a convolutional neural network (CNN) called U-net to extract meaningful features from video frames. Both approaches enabled the automatic detection of flow boiling conditions, such as bubbly, slug, and annular flow, as well as automatic void fraction calculation. Especially CNN demonstrated its ability to deliver fast and dependable results, presenting an appealing substitute to manual feature extraction. The U-net-based CNN was able to segment flow boiling images with a Dice score of 99.1% and classify the above flow regimes with an overall classification accuracy of 91%. In addition, the neural network was able to predict resistance sensor readings from image data and assign them to a flow state with a mean squared error (MSE) < 10^−6^.

## 1. Introduction

Two-phase flow boiling in microchannel heat sinks is one of the most important topics in the field of fluid flow and heat transfer, especially due to the promising application in high heat flux cooling [1,2,3]. It offers significant advantages for the cooling of power electronics, computer chips, laser diodes, and other electronic components due to its large heat transfer surface area and compact design. This approach offers two main benefits, an increased heat transfer coefficient and increased heat dissipation capacity, even at low mass flow rates [4]. Microchannel flow boiling heat transfer is currently one of the most promising approaches for removing significant heat loads from electronic devices [5]. To further improve the flow boiling mechanism, it is important to have a better understanding of the vapor bubble dynamics, the flow regime, and the void fraction inside the microchannel [6]. Typical flow patterns observed in the microchannel during flow boiling are bubbly, slug, and annular flow, as well as the chaotic mixtures and transitions that occur between them [7]. The knowledge of these patterns allows for the derivation of several significant thermal-hydraulic parameters, such as two-phase viscosity and two-phase density, which can be instrumental in forecasting heat transfer coefficients and pressure drop in microchannel cooling devices. The void fraction represents the fraction of the cross-sectional area occupied by the vapor phase compared to the total cross-sectional area [8]. Typically, this void fraction can be determined with high precision by optical investigations at specific locations within the microchannel using high-speed video (HSV) imaging [9]. Alternatively, the flow regime and void fraction are estimated from electrical resistance and impedance measurements, relating resistance and impedance to void fraction and flow regime [10,11]. However, it is a time-consuming task to manually determine flow regimes and void fractions, whether electrically or optically.

For this reason, in response to the rapid progress of computer vision techniques, a few researchers have started to use image processing and machine learning to automatically analyze HSV images of two-phase flows [12]. The use of these tools not only enables the rapid analysis of several thousand HSV image frames in a matter of seconds while maintaining a standardized quantitative approach but also serves as a safeguard against the omission of crucial information due to human error.

In 2011, Hanafizadeh et al. [13] employed basic image processing techniques, including color format conversion, image subtraction, median filtering, and threshold segmentation, to create a binary representation of two-phase flow patterns in the up-riser of airlift pumps. However, the data analysis was still conducted manually, using a ruler, rather than utilizing computer vision (CV) methods. In another study, Singh et al. [14] conducted a more advanced image analysis technique that was performed to generate flow regime maps of flow boiling water in silicon microchannels. The captured HSV images were subjected to sophisticated image processing steps using background removal, cropping, color format conversion, histogram equalization, median filtering, edge detection, contour filling, and binary liquid/vapor region segmentation to determine local void fractions at multiple locations along the microchannel. The void fractions were then used to automatically identify and separate bubbly, slug, and annular flow regimes using predefined void fraction thresholds selected based on careful visual observations.

Very recently, researchers started to apply machine learning techniques to study flow boiling in microchannels and microchannel pin-fins. However, most of the studies focus on the prediction of heat transfer coefficients and pressure drops based on universal consolidated data under the use of artificial neural networks [15,16,17,18,19,20,21,22]. So far, only a handful of studies have used machine learning based on convolutional neural networks (CNNs) to automatically detect bubbles, classify flow regimes, and calculate void fractions from HSV images taken during two-phase flow processes [23,24,25,26].

Kim et al. [23] harnessed the Mask R-CNN to create an automated tool for bubble detection and mask extraction in gas–liquid two-phase flows. Their model was trained with a combination of experimental and synthetic bubbly flow images from upward bubbly flows in expansion pipes [27] and from utilizing the BubGAN algorithm [28]. The trained model reached an average precision of AP_50_ of 98% on unseen test data from the experimental bubbly flow. Additionally, the model was tested on unseen bubble-swarm flows [29] not included in the training set, where it was able to detect 95% of the bubbles. Although the results are remarkable, the model is limited to detecting bubbly flow at low void fraction values. It lacks the ability to detect more complex two-phase flows such as slug and annular flow. The model also does not include flow regime classification.

In another study, Kadish et al. [24] classified vapor quality and flow regimes of vertical two-phase (vapor-liquid) CO_2_ flow images captured at frame rates of 10 fps and 30 fps, respectively, in an 8 mm diameter transparent circular channel using a CNN with ResNet101 for image feature extraction and a deep long short-term memory (LSTM) network to incorporate temporal information of image sequences. The model was trained on a data set of 39,261 manually labeled image frames using cross-entropy loss and the Adam optimization function at a learning rate of 10^−4^, a batch size of 256 for 60 epochs on an NVIDIA® Kepler™ K40 M GPU with 12 GB of GPU accelerator memory. For the flow identification task, there were two output layers in the high-level network architecture called FrameNet and FlowNet. The FrameNet output layer came directly after the CNN, skipping the LSTM network. The FlowNet output layer, on the other hand, came after the LSTM network, using the CNN and the LSTM network for flow regime classification. The authors validated the performance of both flow classification models using five-fold cross-validation on unseen test data, resulting in an accuracy of 91.8% for FlowNet and 92.3% for FrameNet, which in our opinion casts doubt on whether the LSTM network really helps to improve the classification accuracy of flow regime classification.

In the present study, to the best of our knowledge, we are the first to use state-of-the-art image processing techniques and deep learning for binary image segmentation, automatic void fraction calculation, flow regime classification, and RTD sensor signal prediction during two-phase flow boiling in microchannels. For machine learning, we used a CNN based on the U-net architecture [30], using a very limited data set of only 4010 HSV image frames recorded during the flow boiling process in the microchannel as input. The U-net-based CNN classified microchannel flow boiling into 7 flow regimes: bubbly, bubbly-slug, slug, slug-annular with bubbles, slug-annular, annular with bubbles, and annular flow. The RTD sensor signal prediction was intended to be the first step towards automated flow regime detection based on electrical sensor data without cost-intensive optical inspection. The information about the present flow regime during microchannel flow boiling by the automated evaluation of electrical sensor signals from RTDs could pave the way for automated flow regime control in commercial microchannel heat sinks by initiating AI-based control signals to adjust the present flow regime by means of the read-out sensor data. For example, as we have shown in [31], microheaters could be actuated to adjust the flow regime in microchannels via short, low-power heating pulses.

## 2. Materials and Methods

In this section, the experimental setup, the computer vision, and deep learning techniques are described. The computer vision and deep learning code can be downloaded from github.com/shayanjunaidtalat (accessed on 29 April 2024).

### 2.1. Experimental Flow Boiling Apparatus

**Figure 1:**
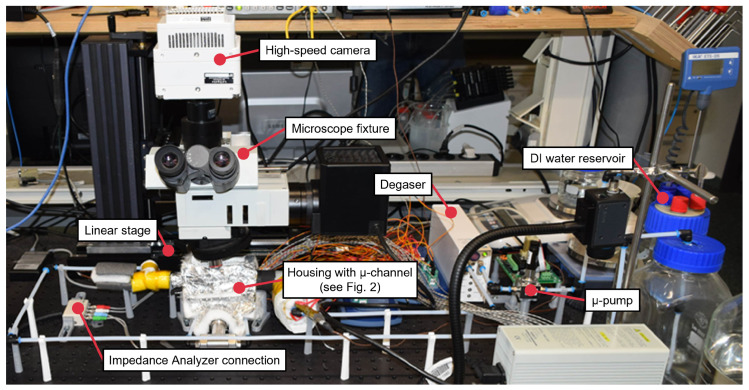
Experimental setup to capture two-phase flow boiling videos and resistance sensor data.

The experimental setup that was used to capture microchannel (MC) two-phase flow boiling videos and sensor data from thin-film platinum RTDs is illustrated in Figure 1. This setup was also used in similar configurations in [10,26,31,32,33,34], where further details about the setup and its application can be found if the reader is interested. The MC with a depth of 0.5 mm, a width of 1.5 mm and a length of 65 mm was milled into a 5 mm × 8 mm × 68.6 mm stainless steel block. The RTDs were manufactured on a Pyrex glass wafer in a clean-room process that is described in detail in [10]. The important components of the setup for the experiments in this paper are a DI water reservoir, a micro pump to supply DI water from the reservoir to the stainless-steel MC, a 3D-printed housing for mechanical fixation and hermetic sealing of the MC, thin-film platinum RTDs on a transparent Pyrex glass lid mounted above the MC, a glass-wool wrapping around the housing for thermal isolation from the environment, heater cartridges inserted at the MC bottom to heat the MC and initiate two-phase flow boiling, a Phantom VEO 410L high-speed camera mounted on a microscope fixture with a 2× magnifying objective to capture the videos of the MC flow boiling process at a frame rate of 2000 fps, a linear stage that moved the microscope fixture with the high-speed camera along the MC to record flow boiling at different MC locations, and an MFIA impedance analyzer from Zurich Instruments to read out the RTD measurements.

**Figure 2:**
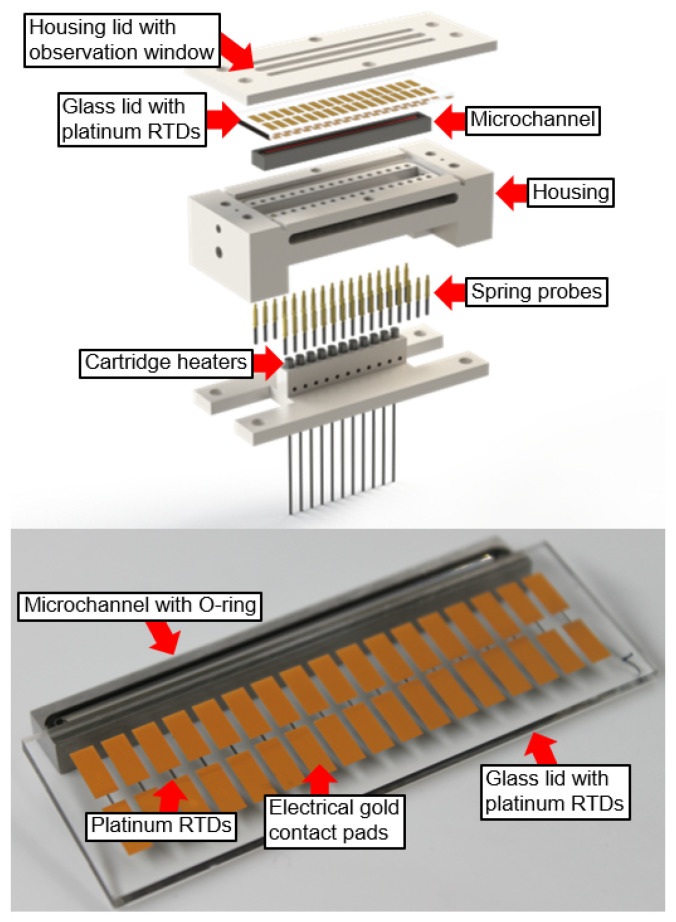
Exploded view of the 3D-printed housing with microchannel (MC), platinum resistance temperature detectors (RTDs), and heating cartridges (**top**) and a closeup of the glass lid with the RTDs (**bottom**).

Figure 2 shows the housing with the MC, the RTDs, and the cartridge heaters in more detail. The housing lid pressed the glass lid with the RTDs on top of the MC in such a way that all RTDs were in direct contact with the flow boiling fluid. The MC was surrounded by an O-ring that ensured a watertight sealing. The boiling process could be observed through the observation window in the middle of the housing lid. The spring probes provided electrical contact to the RTDs via the gold contact pads at each side of the RTDs. The MFIA impedance analyzer was connected via a trigger cable to the high-speed camera, which provided a time-synchronized video and RTD sensor data acquisition. The RTDs detected the temperature-induced resistance changes that were caused by the temperature fluctuations of the flowing fluid ($T_{fluid}$) inside the MC. The resistance/temperature dependency of the RTDs can be described with the following formula:

(1) $$R_{RTD}=\alpha \left(T_{fluid}-T_{0}\right)R_{0}+R_{0},$$

with the measured RTD resistance $R_{RTD}$, the temperature coefficient of resistance $\alpha =2.98\cdot 10^{-3}$°C^−1^, the fluid temperature $T_{fluid}$, the RTD room temperature resistance $R_{0}$, and the room temperature $T_{0}$ [10].

Vapor bubbles passing an RTD structure could be detected by an increase of the measured resistance $R_{RTD}$ due to a temperature increase in $T_{fluid}$ beneath the RTD to the boiling temperature of water at atmospheric pressure (≈100 °C).

### 2.2. Video Data Pre-Processing

**Figure 3:**
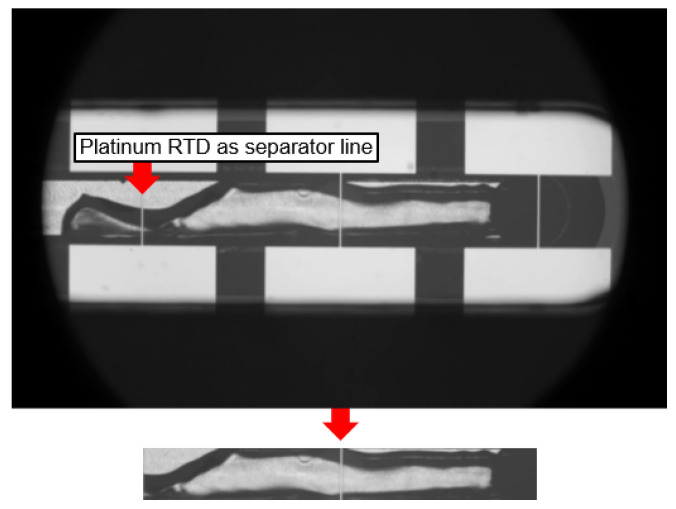
Raw 800 × 1280 pixel image frame of a video of an MC subsection captured with the high-speed camera (**top**). A cropped 112 × 690 pixel version of the image frame after pre-processing, focusing only on the relevant flow boiling pixel area (**bottom**).

Due to the length of the MC of 65 mm, it was not feasible to capture the entire flow boiling phenomenon at once in one video. Therefore, several videos of subsections were recorded along the MC to cover the whole channel length. Figure 3 shows at the top a raw unprocessed video frame of such an MC subsection. Each raw frame of a subsection video underwent a pre-processing pipeline to extract only the meaningful flow boiling pixel data. As shown at the bottom of Figure 3, the pixel area of the flowing fluid inside the MC was considered for further processing, while the rest of the pixel area was discarded. The thin platinum RTDs in Figure 3 were critical for this cropping process. Three vertical platinum RTDs were present in the video frames of each subsection. These platinum RTDs acted as separator lines and were manufactured similarly to the thin-film platinum RTDs for resistance measurements but with a width of only 20 μm to minimize obstruction of the view into the MC. An algorithm was developed to detect these vertical lines and crop the area between the outer two platinum RTD separator lines (ignoring the platinum RTD in the middle) and the MC walls. Therefore, each video represented a subsection covering the entire length of the flow boiling in the MC. This cropping process prevented overlapping pixels of subsection areas and focused solely on the flow boiling in each channel section. Another important reason for the pre-processing is the substantial reduction of computational cost during the application of computer vision and deep learning. The cropping reduced the effective frame size by 92% from 800 × 1280 pixels to 112 × 690 pixels. It is important to be aware of the fact that, in this pre-process, one separator line of a subsection is always part of a neighboring subsection. For example, the outer left channel part next to the left separator line in Figure 3 that is cropped is visible in the neighboring subsection on the left, where this line represents the separator line on the right. The pre-processing algorithm is robust to varying input frame pixel sizes and varying lighting conditions.

### 2.3. Computer Vision for Binary Image Segmentation

**Figure 4:**
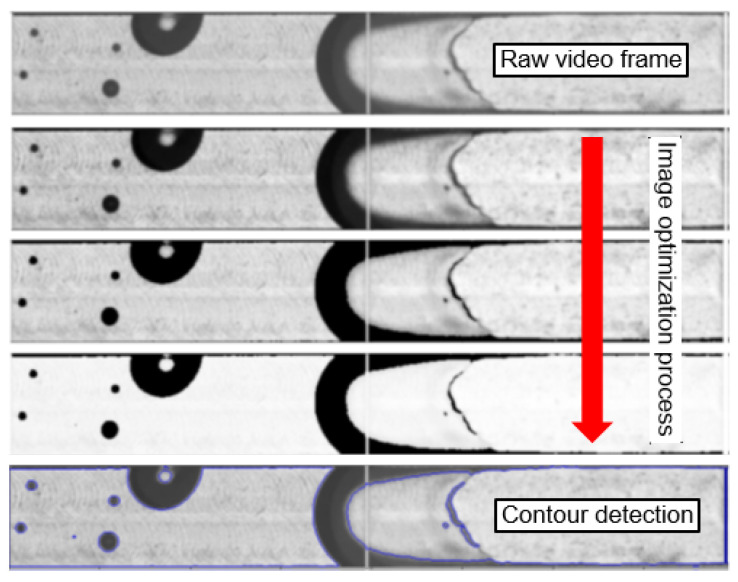
Image optimization process to create initial segmentations from raw video frames using adaptive thresholding, blurring, and gamma correction.

**Figure 5:**
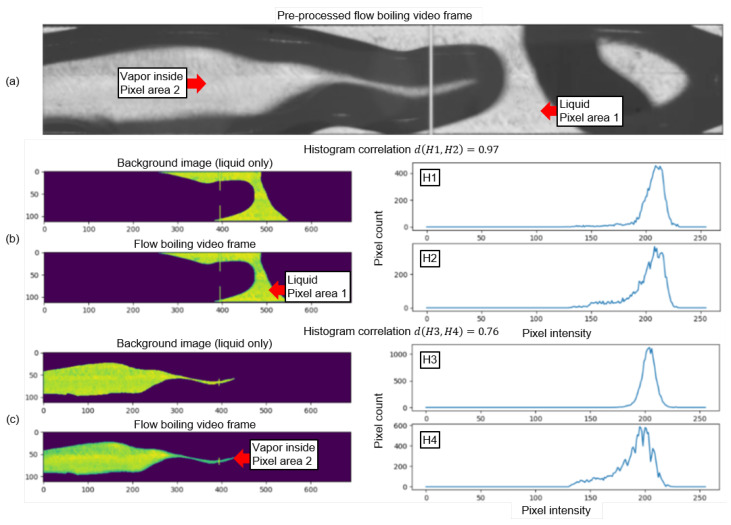
Pixel intensity histogram correlations d(H1,H2) and d(H3,H4) of a flow boiling video frame (**a**) of the liquid contour H2 (**b**) and the contour detected inside the vapor bubble H4 (**c**) with the pixel intensities H1 and H2 of the same pixel areas of the liquid-only background image shown in Figure 6.

**Figure 6:**
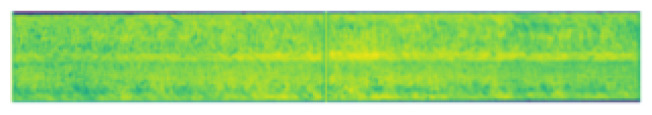
Liquid-only background photo of the MC subsection used in Figure 5 captured at adiabatic conditions before the flow boiling process was initiated.

During MC flow boiling, the fluid prevails in two states of matter: gaseous and liquid. This is tailor-made for the application of binary image segmentation to further reduce the image complexity, bit depth, and memory size of the pre-processed video frames by setting the pixel intensity of liquid areas to 255 (white) and the pixel intensity of vapor areas to 0 (black). In preceding work on computer-vision-based vapor bubble segmentation in two-phase flows, like in [35,36], the vapor bubbles that occurred were small and had regular spherical or elliptical shapes. In this case, the Hough circle transform can be used to detect the presence of vapor bubbles whereas the rest of the section is considered as liquid. However, in our MC, there was a recurring instance of more complex irregular shapes of vapor bubbles that could not be detected by the Hough Transform. Other fundamental challenges were textured lines at the MC background, as well as differentiating the inside of vapor bubbles from the liquid part. The textured MC background was removed by an image optimization process (Figure 4) that used adaptive thresholding, blurring, and gamma correction. This process created an initial segmentation that did not yet differentiate between vapor bubbles inside liquid sections. A contour detection (Figure 4) and histogram comparison (Figure 5) finally enabled the differentiation of pixel areas inside the vapor from pixel areas of the liquid. This was performed by correlating the pixel intensity histograms of these pixel areas with the same pixel areas of a single liquid-only background photo (Figure 6) of the same MC subsection captured at adiabatic conditions before the two-phase flow boiling videos were recorded. The histograms were correlated utilizing the magnitude of the Pearson correlation coefficient *d*, which takes values between 1 (full correlation) and 0 (no correlation). It is defined as

(2) $$d\left(x,y\right)=|\frac{\sum_{i=1}^{n}\left(x_{i}-\bar{x}\right)\left(y_{i}-\bar{y}\right)}{\sqrt{\sum_{i=1}^{n}{\left(x_{i}-\bar{x}\right)}^{2}\sum_{i=1}^{n}{\left(y_{i}-\bar{y}\right)}^{2}}}|,$$

where

$$\begin{array}{ccc} \bar{x}=\frac{1}{n}\sum_{i=1}^{n}x_{i}, & \; & \bar{y}=\frac{1}{n}\sum_{i=1}^{n}y_{i}, \end{array}$$

with the histogram size *n*, the individual histogram data points $x_{i},y_{i}$, and the histogram mean values $\bar{x},\bar{y}$.

Figure 5 compares pixel intensity histograms of a liquid contour (*H*2 in Figure 5b) and a contour detected inside a vapor bubble (*H*4 in Figure 5c) of a flow boiling video frame (Figure 5a) with the pixel intensity histograms (*H*1 and *H*3 respectively) of the same pixel areas of a liquid-only background photo and states the histogram correlations d(H1,H2) and d(H3,H4). The pixels that were part of the contours and that were therefore used as data sets for the pixel intensity histograms are highlighted in green-yellow. The pixel areas in dark purple are not included in the respective histogram data sets. The liquid-only background images in Figure 5 highlight and discard the same pixel areas as the flow boiling video frames, even when the whole area was composed of liquid. This was important so that the same pixel areas were always compared with each other. The histogram correlation of pixel area 1 was d(H1,H2)= 0.97, and the histogram correlation of pixel area 2 was d(H3,H4)= 0.76. After analyzing several thousand flow boiling video frames with a histogram correlation sweep, it was found that for a histogram correlation of d≥0.85, most of the compared contour pixel areas were liquid. Respectively, below this value, most of the pixel areas were vapor inside areas. Therefore, 0.85 was set as threshold value for labeling contours as liquid. As a last step of the binary segmentation process, all pixel intensity values of pixels labeled as liquid were set to 255 (white). All other pixel intensities were labeled as vapor inside and set to 0 (black). The cross-sectional void fraction, which represents the proportion of the area taken up by the vapor phase in relation to the total cross-sectional area was automatically calculated for each binary segmentation frame as follows:

(3) $$\mathrm{Void}\;\mathrm{fraction}=\frac{\mathrm{Pixels}\;\mathrm{with}\;a\;\mathrm{pixel}\;\mathrm{intensity}\;\mathrm{of}\;0}{\mathrm{Total}\;\mathrm{No}.\;\mathrm{of}\;\mathrm{MC}\;\mathrm{subsection}\;\mathrm{pixels}}$$

The computer vision algorithm saved all input two-phase flow boiling video frames, and the resulting binary segmented output frames in designated folders on the computer hard disk.

### 2.4. Deep Learning for Binary Image Segmentation, Flow Regime Classification and RTD Data Prediction

Using classical computer vision methods to distinguish vapor from liquid areas in the MC gave good results (see Section 3), but some pixel areas were still misclassified as liquid or vapor despite all efforts to fine-tune the parameters of adaptive thresholding, blurring, gamma correction, and histogram correlation. This led to the question of whether or not deep learning would help to improve the results of binary image segmentation. The first step was to manually inspect the binary segmentation directory for mis-segmented images, which were then manually corrected using a custom-built manual segmentation function. A training and a test data set were then created in an 80:20 ratio. For this purpose, 4010 pre-processed input video frames and manually corrected output binary segmentation frames were selected from randomly chosen sections of the respective directories to ensure that data from all recorded videos were selected in both the training and test data sets. The Dice coefficient below was used as the loss function:

(4) $$\mathrm{Dice}\;\mathrm{coefficient}=\frac{2\cdot \mathrm{Overlapping}\;\mathrm{pixel}\;\mathrm{area}}{\mathrm{Total}\;\mathrm{No}.\;\mathrm{of}\;\mathrm{pixels}}$$

The overlapping pixel area is the area of overlap between the predicted binary segmentation of the model and the training or test data, respectively. The Dice coefficient is one of the most common loss functions used for image segmentation [37].

The deep learning model used for binary segmentation is a modified form of the U-net architecture published by Ronneberger et al., 2015 [30]. The following modifications were made to the original model:

- Input layer image channels were set to 3, as the video frames are in RGB format.
- Output layer image channels were set to 1 since the masked images obtained were in greyscale form, with 0 denoting pixels identified as vapor and 1 denoting pixels identified as liquid.
- The kernel size of the first convolutional layer was set to 7 × 7 with a rectifier linear unit (ReLU) activation function. This change helped to process larger images with half the computational cost.

**Figure 7:**
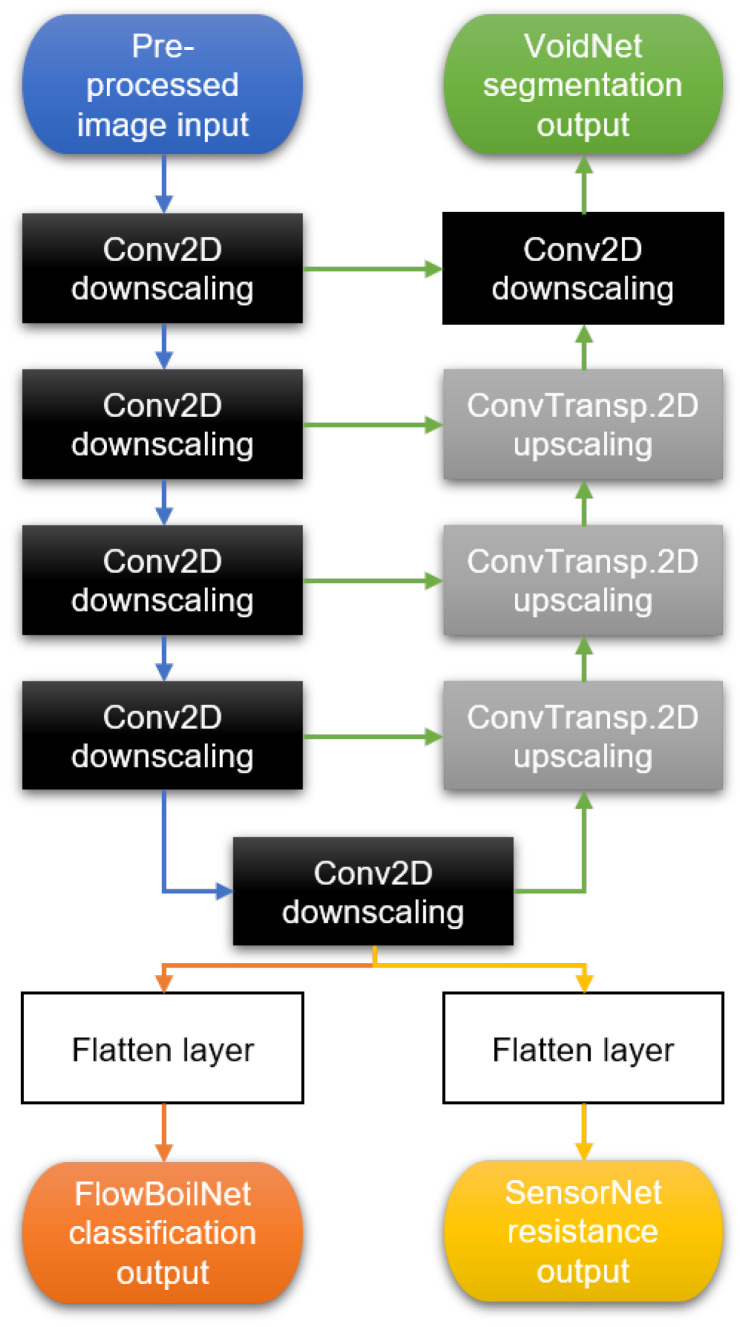
Simplified network architecture of VoidNet for binary image segmentation (green), FlowBoilNet for flow regime classification (orange), and SensorNet for RTD signal prediction illustrated in a single flowchart. The arrow colors visualize the paths taken by each model, with the blue path being taken by all models.

As with the original U-net model, concatenated skip links were used to ensure feature reusability. The DL model for binary segmentation was trained using Dice coefficient loss and the Adam optimization function [38] at a learning rate of 10^−5^ and a batch size of 32 for 50 epochs on a 6 GB NVIDIA GTX3060 laptop GPU and is referred to as VoidNet in this paper. The simplified network architecture for all DL models is illustrated as a flowchart in Figure 7.

Some modifications were made to the deep learning model described above for flow regime classification (designated as FlowBoilNet) and RTD sensor data prediction (designated as SensorNet). As shown in Figure 7, FlowBoilNet and SensorNet use only the Conv2D downscaling path to classify flow regimes and predict RTD signals from extracted downscaled image features. However, the randomized selection of training and test data remained constant at an 80:20 ratio.

**Figure 8:**
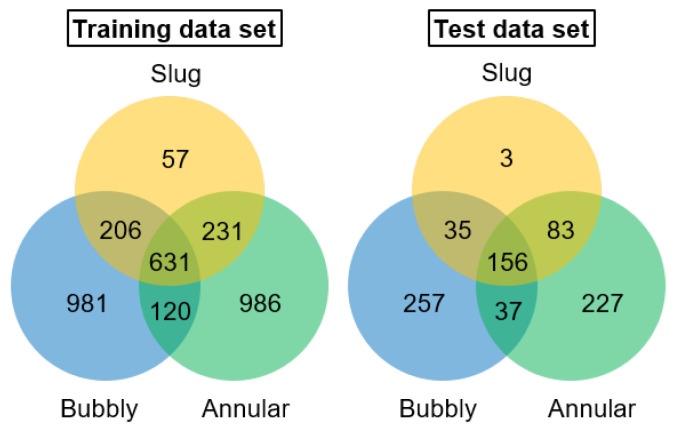
Training and test data distribution for FlowBoilNet.

For the flow regime classification, the 4010 pre-processed video frames were manually labeled as expected output according to the current flow regime in the form of 3 × 1 tensors. Figure 8 shows the labeled training and test data distribution for FlowBoilNet. The model output layer of the modified U-net model was adapted accordingly to output the predicted flow regime in the form of 3 × 1 tensors. All possible tensors are listed in Table 1. The loss function was changed to cross-entropy, one of the most common loss functions for classification problems [39], and the learning rate was set to 10^−4^.

For RTD sensor data prediction, 12,340 video frames were automatically labeled with the corresponding measured RTD resistance and split 80:20 into training and testing data. A trigger cable connecting the impedance analyzer and the high-speed camera synchronized the video frames with the measured RTD data. The output layer of the modified U-net model was adjusted to output real numbers representing the predicted RTD resistance. The mean square error (MSE) was used as a loss function to solve the linear regression problem. An overview of all hyperparameters is shown in Table 2.

## 3. Results and Discussion

**Figure 9:**
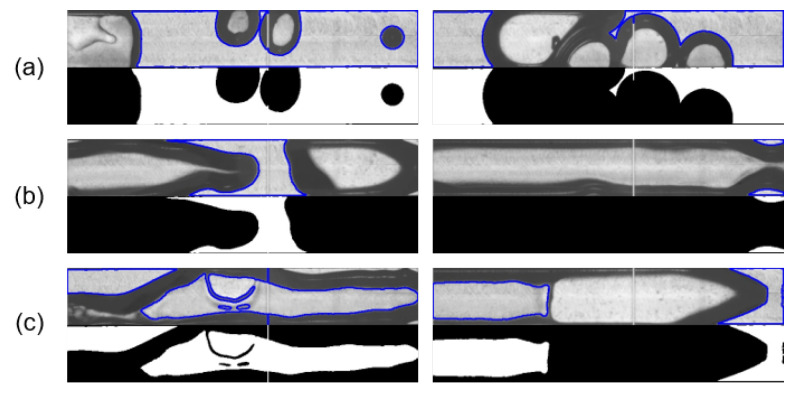
Results of the binary image segmentation using CV, for separated and overlapping vapor bubbles (**a**), slug and annular flow (**b**), and incorrectly segmented HSV images (**c**).

Segmented HSV image frames using the CV method described in Section 2.3 are shown in Figure 9. The majority of all video frames could be correctly segmented in binary using CV. From separated and overlapping bubbles of different shapes and sizes (Figure 9a) to slug and annular flow (Figure 9b), the CV method was able to successfully segment most of the HSV images even for complex vapor/liquid mixtures. However, approximately 15% of all frames were incorrectly segmented. This was mainly because the image processing could not always distinguish the inside of the gas bubbles from the liquid segments, as shown in Figure 9c, despite careful fine-tuning of the histogram correlation *d* to a liquid-only background image (Figure 6).

**Figure 10:**
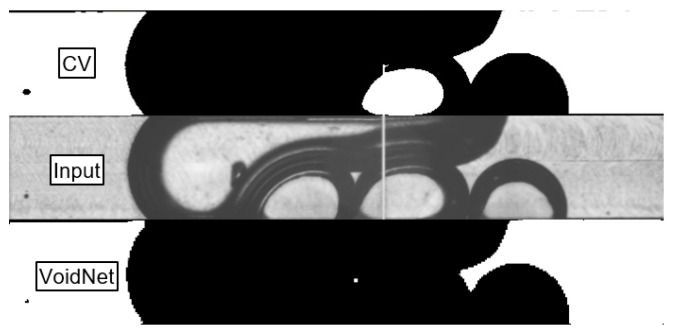
Binary segmentation of VoidNet after 50 epochs (**bottom**) compared to the binary segmentation of the CV method (**top**) for the same input frame (**middle**).

Figure 10 shows an input frame from the DL test data set and the corresponding prediction of the trained VoidNet model after 50 epochs compared to the binary segmentation of the CV method. It can be seen that VoidNet reliably distinguishes between liquid and gaseous regions. Overall, the VoidNet model achieves a Dice score of 99.1% after 50 epochs in both the training and test runs, significantly outperforming the CV method.

**Figure 11:**
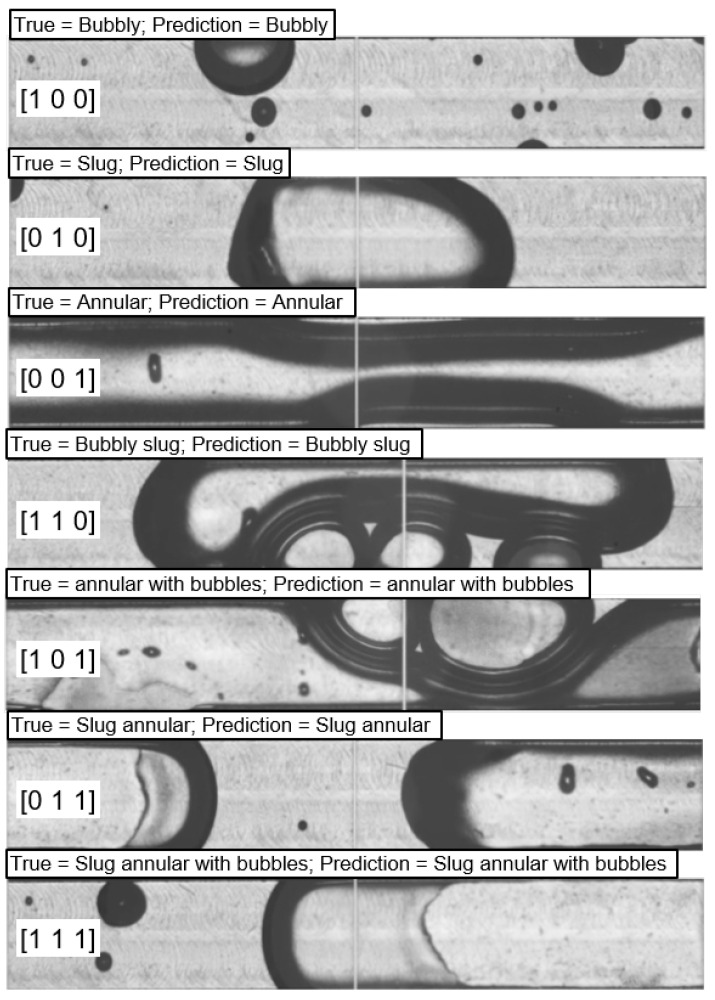
FlowBoilNet classification result for HSV images covering all of the flow regimes listed in Table 1.

**Figure 12:**
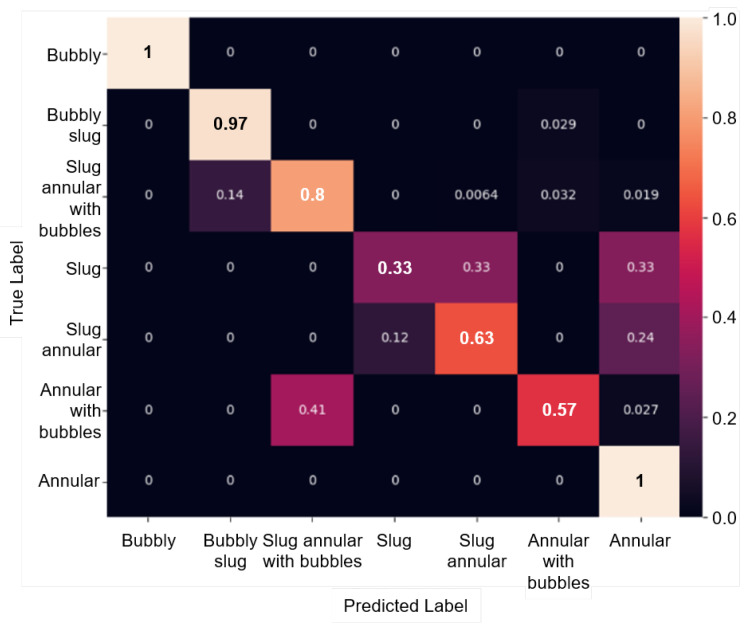
Normalized confusion matrix of the 50th epoch for flow regime classification of the training data set using FlowBoilNet.

Figure 11 shows the classification result of FlowBoilNet for all flow regimes given in Table 1. FlowBoilNet was able to classify flow regimes with an overall classification accuracy of 91%. The normalized confusion matrix shown in Figure 12 illustrates the ratio between the true and predicted values of the trained FlowBoilNet model for all classified flow rates (Table 1), with the individual flow regime accuracies shown in the diagonal from top left to bottom right. A normalized confusion matrix is a good way to visualize the accuracy of each class. This is especially helpful in the case of imbalanced data sets, as the overall accuracy of 91% does not reveal classification biases across classes. In Figure 12, it can be seen that for some flow rates, like bubbly, bubbly slug, and annular flow, FlowBoilNet performs very well and classifies the flow regime correctly with an accuracy between 97 to 100%. However, for other flow rates, the FlowBoilNet model does not perform as well, and the classification accuracy even goes below 50% like in the case of slug flow.

This difference in classification accuracy of the trained deep learning model can be described by the imbalanced data distribution of the test and training data set shown in Figure 8. The accuracy of flow regime classification decreases significantly as the amount of training data for each flow regime decreases. For example, the slug flow regime is the least represented with 57 frames during the training run. Accordingly, the slug flow regime has the lowest accuracy at 33%, followed by annular with bubbles at 57% and slug annular at 63%. However, the normalized confusion matrix demonstrates that flow regimes are merely confused with very similar neighboring flow regimes; e.g., the slug flow is only confused with the very similar slug annular and annular flow.

**Figure 13:**
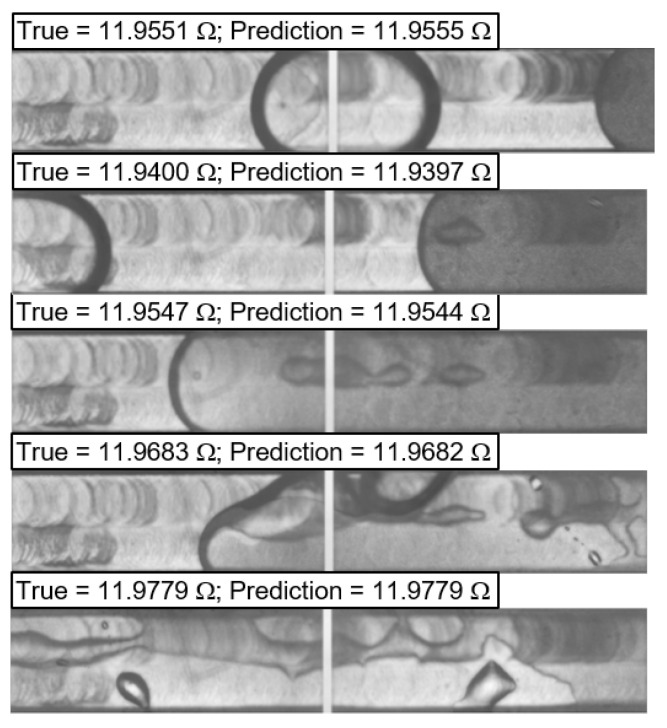
SensorNet RTD signal prediction result for different microchannel flow boiling patterns.

The prediction of the RTD data is shown in Figure 13 as an example of different kinds of flow patterns. In general, an impressive loss of <10^−6^ was achieved in the training run. However, two divergence peaks occurred in the test run, indicating problems with overfitting or a lack of generalization.

Overall, the trained deep learning models produced reliable results thanks to the state-of-the-art U-net architecture, which was able to extract meaningful representations from the microchannel flow boiling video frames. However, for better results, the use of more training data could further improve the accuracy of the model. Increasing the training data set is one of the most effective ways to address the lack of generalization. The experimental setup is subject to different lighting conditions and slightly tilted microchannel positions. Training the model on a variety of HSV image frames will result in more accurate predictions, especially between very similar flow regimes such as slug, slug-annular, and annular flow.

## 4. Conclusions

In conclusion, our study pioneers the application of image processing and deep learning techniques in microchannel two-phase flow boiling. In particular, the U-net-based CNN architecture was used to automatically identify flow regimes and void fractions in microchannel flow boiling. The approach exhibited a high classification accuracy of 91%, demonstrating the efficiency of CNN in accurately discriminating flow regimes such as bubble, slug, and annular flow. In addition, our exploration of real-time RTD sensor signal prediction marks a significant step toward the imageless prediction of two-phase flow in microchannels. The promising results, including a mean squared error (MSE) < 10^−6^ in predicting RTD sensor readings, suggest the potential for automated flow regime control. While our trained models demonstrated reliability, it is critical to recognize the need for a more extensive training data set to account for variations in lighting conditions and microchannel positions. Nevertheless, the implemented approaches, executed on a standard 6 GB NVIDIA laptop GPU using Python, demonstrate adaptability for broader applications beyond microchannel flow boiling. Looking to the future, this research envisions the integration of these automated techniques into microchannel heat sink control systems, potentially revolutionizing the field by leveraging artificial intelligence for improved operational efficiency and control. The results presented here lay the foundation for future advances in the seamless automation of two-phase flow boiling processes.

## Figures and Tables

**Table 1:** Tensor output of the flow regime classification model FlowBoilNet.

Flow Regime	3 × 1 Output Tensor
Bubbly	[1 0 0]
Slug	[0 1 0]
Annular	[0 0 1]
Bubbly slug	[1 1 0]
Annular with bubbles	[1 0 1]
Slug annular	[0 1 1]
Slug annular with bubbles	[1 1 1]

**Table 2:** Training hyperparameters for VoidNet, FlowBoilNet and SensorNet.

Hyperparameter	VoidNet	FlowBoilNet	SensorNet
Loss	Dice coefficient	Cross entropy	MSE
Optimizer	Adam	Adam	Adam
Learning rate	10^−5^	10^−4^	10^−6^
Batch Size	32	32	32
Epochs	50	50	50

## Data Availability

Data set available on request from the authors.

## Abbreviations

The following abbreviations are used in this manuscript:

CNN	Convolutional neural network
CV	Computer vision
DL	Deep learning
HSV	High-speed video
LSTM	Long short-term memory
MSE	Mean square error
ReLU	Rectifier linear unit
RTD	Resistance temperature detector
